# MicroRNA-572 Improves Early Post-Operative Cognitive Dysfunction by Down-Regulating Neural Cell Adhesion Molecule 1

**DOI:** 10.1371/journal.pone.0118511

**Published:** 2015-02-13

**Authors:** Xiya Yu, Shupeng Liu, Jinbao Li, Xiaohua Fan, Yuanjie Chen, Xiaoying Bi, Shanrong Liu, Xiaoming Deng

**Affiliations:** 1 Department of Anesthesiology, Changhai Hospital, Second Military Medical University, Shanghai, 200433, China; 2 Clinical Research Center, Changhai Hospital, Second Military Medical University, Shanghai, 200433, China; 3 Department of Neurology, Changhai Hospital, Second Military Medical University, Shanghai, 200433, China; National University of Singapore, SINGAPORE

## Abstract

Post-operative cognitive dysfunction (POCD) is a commonly-seen postoperative complication in elderly patients. However, the underlying mechanisms of POCD remain unclear. miRNAs, which are reported to be involved in the pathogenesis of the nervous system diseases, may also affect POCD. In this study, miRNA microarray technology was used to analyze the circulating miRNA expression profile of POCD patients. Among the altered miRNAs, miR-572 had the greatest decrease, which was also verified *in vivo* in rat POCD model. Further analysis found that miR-572 could regulate the expression of NCAM1 in the hippocampal neurons and interfering miR-572 expression could facilitate the restoration of cognitive function *in vivo.* Moreover, clinical correlation analysis found that the miR-572 expression was associated with the incidence of POCD. Collectively, miR-572 is involved in the development and restoration of POCD and it may serve as a biological marker for early diagnosis of POCD.

## Introduction

Human cognition is an important part of neuroscience with intriguing findings and immense implications recently [1]. With development of molecular technology, it is possible to identify molecules that correlated with human cognitions. It has been reported that molecular alterations including single nucleotide polymorphism are involved in human cognition through their roles in the nurture and maintenance of the central nervous system (CNS) [2].

Post-operative cognitive dysfunction (POCD) is a commonly-seen postoperative complication in elderly patients [3, 4]. POCD is especially common after cardiac surgery with a cardiopulmonary bypass [5]. However, it has been reported recently that 25.8% of patients aged over 65 years old developed POCD at 1 week after non-cardiac surgery under general anesthesia, with some having a long duration of cognitive dysfunction and even developing dementia [6], and the incidence of POCD dropped to 12.7% at 3 months after surgery [7, 8]. These findings suggest that the early postoperative nervous damage can lead to cognitive dysfunction, and the body simultaneously initiates the protective mechanism for self-repair, thus improving or restoring the cognitive function in some patients. Therefore, exploring the mechanism of postoperative cognitive function is of great significance for improving the prognosis of patients and identifying therapeutic targets for restoration of cognitive function.

MicroRNAs (miRNAs) are widely distributed in eukaryotes and regulate the translation and transcription of target genes by degrading target mRNAs or inhibiting the translation of target genes [9]. Recently, studies have shown that miRNAs are also involved in the pathogenesis of the nervous system diseases [10, 11]. Multiple abnormally expressed miRNAs were found involved in the occurrence of Huntington's disease, a hereditary neurodegenerative disease and that the miRNA expression levels had a certain degree of synergy with the development of the disease [12]. Another study reported that miRNAs also participated in the nervous system development and nervous system damage repair [10]. The abnormal expression of multiple miRNAs was also reported to be associated with the occurrence of neural tube defects [13], abnormal expression of miR-219 led to *N-methyl-D-aspartate* (NMDA) receptor- associated neurobehavioral disorders [11], and abnormal expression of miR-124 was observed in Alzheimer's disease [14].

Recent studies have found that the miRNAs in the circulating peripheral blood are similar to the miRNAs in tissues [15, 16]. As miRNAs are stable in the peripheral blood and it is convenient to collect peripheral blood samples, peripheral blood miRNAs are widely used as diagnosis and prognosis markers for tumors and a variety of other diseases [16, 17]. In the present study, we used microarray technology to detect the miRNA expression profile in peripheral blood and found that the miR-572 expression was significantly altered. Experiments using the POCD model in aging rats found that miR-572 was specifically expressed in the hippocampus area. Reduced expression of miR-572 significantly improved the cognitive function of rats. This suggests that after the occurrence of POCD, the restoration of cognitive function was promoted by the nervous tissues down-regulating the expression of miR-572 and, subsequently, up-regulating the expression of NCAM1. In addition, changes in the plasma expression levels of miR-572 could reflect the progress of POCD.

## Materials and Methods

### Samples

Blood samples were obtained from 38 POCD patients including 29 cases with restoration of cognitive function 3 month after surgery and 9 cases without (S1 Table) in the Department of Anesthesiology at the Changhai Hospital from October 2011 to October 2012.

Inclusion criteria were age >60, surgery for knee or hip replacement under spinal anesthesia, American Society of Anesthesiologists physical status (ASA) 1–3, and patient consent. Exclude criteria were >85, ASA physical status >3, neurological disorders, stroke or other affective central nervous system disease, history of serious psychiatric illness, long-term use of psychoactive substances, multiple trauma and the presence of head injury[18]. Demographic information such as age and gender, co-morbidities, ASA physical status, hemoglobin, hematocrit and type of anesthesia (general or subarachnoid) were recorded on all patients.

Cognitive function was assessed on the day before the surgery and on the 7^th^ and 30^th^ postoperative day, using the MMSE test combined with the Self-Rating Anxiety Scale (SAS) and Self-Rating Depression Scale (SDS) and the American Psychiatric Association Diagnostic and Statistical Manual (DSM-IV). Reduction of MMSE score by ≥ 2 points on the 7^th^ postoperative day compared with preoperative day was considered evidence of cognitive decline [19, 20]. The SAS/SDS were used to exclude severe anxiety and depression [21].

All samples collection was performed with the patients' consent, and signed informed consent was obtained. The study was approved by the Ethics Committee of Changhai Hospital Second Military Medical University. The peripheral blood samples (5 ml) were collected into the anticoagulant tubes at 1 day before surgery, 24 h after surgery, and 7 days after surgery and were centrifuged (4°C, 820 rpm, 10 minutes); the supernatant was stored at—70°C until testing.

HT-22 cells, a widely used hippocampal neuronal cell line derived from parent HT4 cells, were maintained in Dulbecco's modified Eagle's medium (DMEM) supplied with 10% FBS, as previously described [22].

### Microarray

miRNA microarray analyses were performed by Shanghai Biotech Corp. using blood from POCD patients. Briefly, 3 patients (Table 1, P # 2, P # 14, and P # 25) who were clinically diagnosed with POCD 7 days after surgery and who presented with an improvement of cognitive function 3 months later were randomly selected. Then, the preoperative and 24h postoperative peripheral blood samples from these three patients were used for the miRNA microarray analyses using Agilent human miRNA array. The data of microarray analyses was submitted to ArrayExpress with accession number E-MTAB-3187.

**Table 1:** Clinical information of 3 cases of POCD.

Patients NO.	Age	Gender	BMI	ASA	Previous psychoactive drugs	Surgery	Anesthesia method	Postoperative analgesia	MMSE pre	MMSE post
2	71	F	24.65	II	No	Right knee resurfacing arthroplasty	Spinal anesthesia	Sufentanil	30	25
14	67	F	26.22	II	No	Right total hip arthroplasty	Spinal anesthesia	Sufentanil	27	22
25	69	F	23.80	II	No	Left knee resurfacing arthroplasty	Spinal anesthesia	Sufentanil	28	24

ASA: American Society of Anesthesiologists physical status; MMSE: Mini Mental State Examination; BMI: Body Mass Index; Pre: Preoperation; Post: postoperation 7 days.

### Quantitative real-time PCR

Total RNA was isolated from HT-22 cell lines and clinical samples with TRIzol reagent according to the manufacturer’s protocol (Invitrogen, Carlsbad, CA). Reverse transcription reactions were conducted with oligo (dT)18 primers and random primers according to the instructions of the manufacturer of the M-MLV Reverse Transcriptase kit (Invitrogen). MiRNAs reverse transcription was performed using miRcute miRNA First-strand cDNA Synthesis kits (TIANGEN) according to the manufacturer’s instructions. Real-time PCR was performed with SYBR Premix Ex TaqTM (TaKaRa) using the StepOnePlus Real-Time PCR system (Applied Biosystems, Foster City, CA). The primers used in these assays are listed in Table 2. The gene expression levels were calculated relative to the expression of β-actin or U6 in cell lines or clinical samples using the 2^-ΔΔCt^ method.

**Table 2:** Primers Sequence.

Name	Sequence
miR-572	5' GTCCGCTCGGCGGTGGCCCA3'
U6	5' CAAATTCGTGAAGCGTTCCATAT 3'
Mouse NCAM1	
Sense	5' GGGCAAAGACATGGAGGAGG 3'
Antisense	5' CGCTGGCTTGGCTTCTGACT 3'
Mouse β-actin	
Sense	5'CCTGTATGCCTCTGGTCGTA3'
Antisense	5' CCATCTCCTGCTCGAAGTCT 3'
Mouse NCAM1 UTR WT	
Sense	5' TTGTTTACAGTGGCTCTATCCC 3'
Antisense	5' GGCTTAGCGTCCTCTTCATC 3'
Mouse NCAM1 UTR mut	
Sense	5' AGATTTCAAGGCAAGAGCGCG 3'
Antisense	5' GGCTTAGCGTCCTCTTCATC 3'
pLKO-anti-572	
Sense	5'CCGGTCAGGCGAGCCGCCACCGGGTCTCGAGACCCGGTGGCGGCTCGCCTGTTTTTG3'
Antisense	5'AATTCAAAAACAGGCGAGCCGCCACCGGGTCTCGAGACCCGGTGGCGGCTCGCCTGA 3'
pLKO-anti-mock	
Sense	5'CCGGTTCTCATCTGATTACGAGTGTGTGATCTCGAGATCACACACTCGTAATCAGATGAGATTTTTG 3'
Antisense	5'AATTCAAAAATCTCATCTGATTACGAGTGTGTGATCTCGAGATCACACACTCGTAATCAGATGAGAA3'

### Western blotting analysis

Total soluble proteins (100 μg) extracted from the HT-22 cells were resolved in 10% SDS-polyacrylamide gels and transferred electrophoretically to a polyvinylidene fluoride membrane. Blots were blocked with 5% skim milk, followed by incubation with NCAM rabbit polyclonal antibody (1:300, Abcam.) or β-actin (Cell Signaling). Blots were then incubated with goat anti-mouse or anti-rabbit secondary antibody (Santa Cruz Biotech. Inc.) and visualized by enhanced chemiluminescence.

### Establishment of the POCD animal model

The aged male rats (≥ 18 months; Wistar, n = 60) were purchased from the Transgenic Animal Research Center of the Second Military Medical University. All mice were maintained in a pathogen-free facility and used in accordance with the the Animal Research: Reporting of In Vivo Experiments (ARRIVE) guidelines. The study was approved by the Ethics Committee of Changhai Hospital Second Military Medical University. According to the method described previously, the aged rats (≥ 18 months; Wistar) were anesthetized by inhalating 1.5% isoflurane and underwent a splenectomy to establish the POCD model which was used widely to mimic POCD in elderly human patients [23–25]. The study was divided into 4 groups (n = 15): group A did not receive any treatment, group B received a splenectomy with no postoperative treatment, group C received a splenectomy and 3 days of continuous injection of the miR-572 inhibitor expression vector (pLKO-anti-572) via a lateral ventricular micropump starting at 24 h after surgery, and group D received a splenectomy and 3 days of continuous injection of the control vector (pLKO-anti-mock) via a lateral ventricular micropump starting at 24h after surgery. According to the literatures [26], an intraventricular stereotactic surgery was performed to place the micropump, the miR-572 inhibitor (pLKO-anti-572) and the control (pLKO-anti-mock) were injected into the lateral ventricle at 1 μg/μl/h for 3 consecutive days. The animals were euthanized by anesthetizing with a lethal dose of Nembutal as previously reported to minimize suffering [23].

### Water maze test

The rats were trained for the water maze at 1 day before surgery, and the water maze test was performed 24 h and 7 days after the surgery; and the subsequent data were used as the learning and memory score [23, 27].

### In situ hybridization

Probes of pri-miR-572 with a digoxin label were synthesized for in situ hybridization (ISH). ISH was performed on sections of tissues as previously reported. In situ hybridization is a necessary experimental complement to microRNA (miRNA) expression profiling in the human brain.

### Statistical analysis

Student’s t-tests were used to compare two groups unless otherwise indicated (χ2 test). Categorical data were analyzed using Fisher’s exact test, and quantitative variables were analyzed using t-tests or Pearson’s correlation test. *P*<0.05 was considered statistically significant.

## Results

### Changes in plasma miRNA expression profiles before and after POCD occurrence

**Fig 1:**
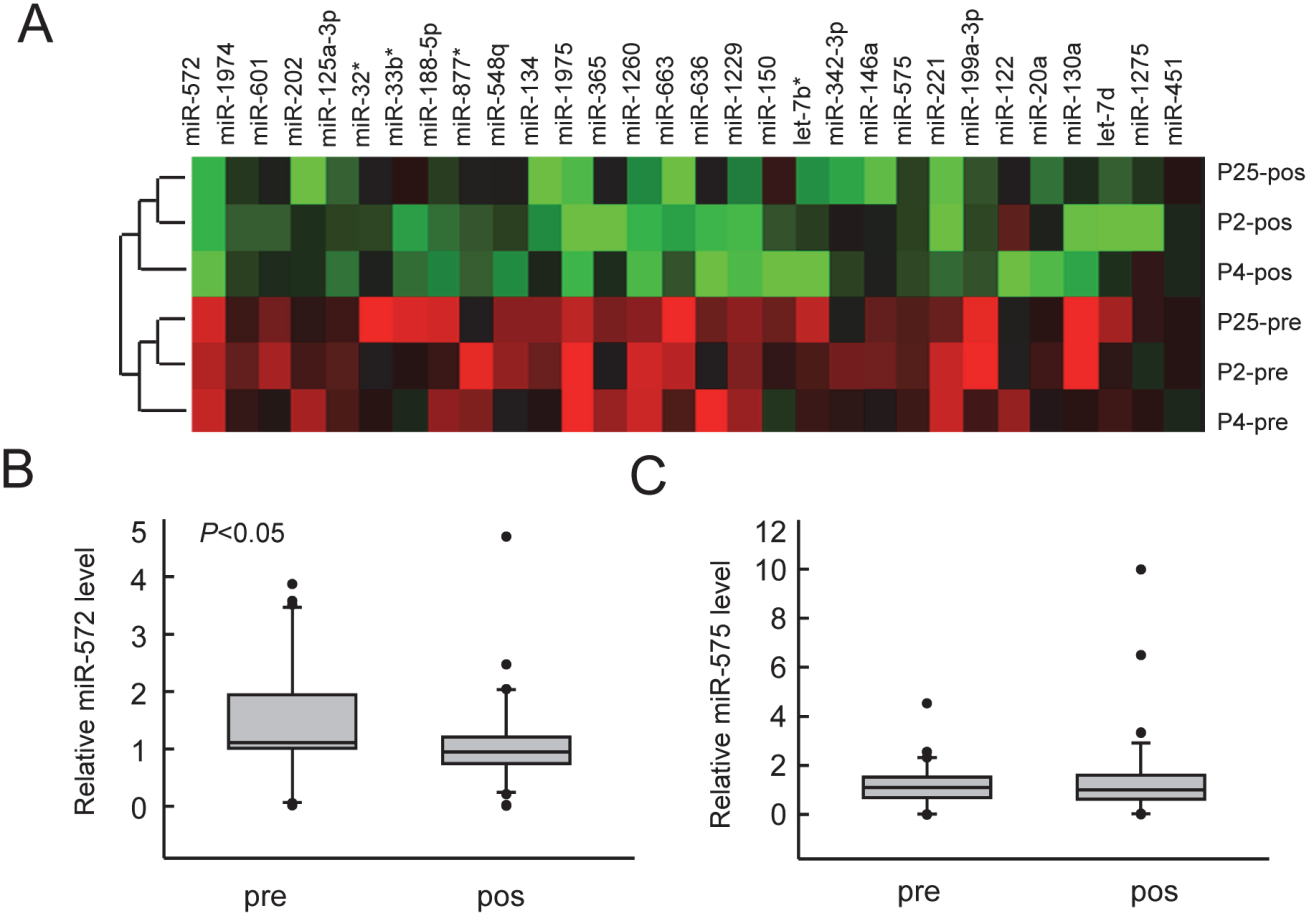
miR-572 levels were decreased in the peripheral blood of POCD patients. A. Microarray results of peripheral blood miRNAs for 3 POCD cases and heat map of cluster analysis for postoperatively downregulated miRNAs. B and C. Changes in the expression of miR-572 and 575 in the peripheral blood of 38 cases of POCD, which were detected using real-time quantitative PCR. pre, preoperation; pos, postoperation.

To screen microRNA associated with cognitive function restoration in patients with POCD, the circulate miRNA expression profiles were analyzed using microRNA microarray as mentioned in the Method. The microarray results showed that, compared with the preoperative microRNA expression profile, there were a large number of abnormally down-regulated circulating miRNA molecules in the peripheral blood 24 h after surgery (Table 3). Cluster analysis found that many microRNAs were abnormally down-regulated, suggesting that specific microRNA expression changes are associated with the restoration process in elderly POCD patients (Fig. 1A). Among these abnormally down-regulated microRNA molecules, the changes of miR-572 and miR-575 were the most significant compared with the preoperative levels, being reduced by 8- and 4-fold, respectively. Real-time quantitative PCR was used to detect the miR-572 and miR-575 expression levels in the plasma samples of 38 POCD patients. The results showed that after development of reduced cognitive function, the miR-572 expression was significantly decreased (Fig. 1B), while the fold change and trend of miR-575 expression were not stable and had no statistical significance (Fig. 1C). Therefore we chose miR-572 as a target molecule in the following studies.

**Table 3:** miRNAs downregulated in the peripheral blood of POCD patients after surgery.

miRNA	Fold change	*P*	Chr^1^	MiRbase accession No.	start	stop	strand
miR-572	8.61±2.30	0.00	chr4	MIMAT0003237	11370519	11370530	-
miR-575	4.13±1.84	0.03	chr4	MIMAT0003240	83674568	83674552	+
miR-663	3.80±2.90	0.05	chr20	MIMAT0003326	26188857	26188846	+
miR-1975	3.20±0.14	0.00	chr7	MIMAT0009450	1.49E+08	1.49E+08	-
miR-1260	2.93±0.64	0.01	chr14	MIMAT0005911	77732578	77732591	-
miR-221	2.90±0.60	0.01	chrX	MIMAT0000278	45605671	45605654	+
miR-134	2.86±2.24	0.05	chr14	MIMAT0000447	1.02E+08	1.02E+08	-
miR-1229	2.54±0.34	0.00	chr5	MIMAT0005584	1.79E+08	1.79E+08	+
miR-636	2.43±1.14	0.07	chr17	MIMAT0003306	74732614	74732603	+
miR-150	2.36±2.14	0.13	chr19	MIMAT0000451	50004078	50004062	+
miR-365	2.36±1.85	0.10	chr16	MIMAT0000710	14403197	14403218	-
let-7d	2.34±1.13	0.04	chr9	MIMAT0000065	96941127	96941144	-
miR-342–3p	2.28±0.75	0.04	chr14	MIMAT0000753	1.01E+08	1.01E+08	-
miR-199a-3p	2.27±0.68	0.04	chr1	MIMAT0000232	1.72E+08	1.72E+08	+
miR-202	2.17±0.93	0.02	chr10	MIMAT0002811	1.35E+08	1.35E+08	+
miR-188–5p	2.02±0.25	0.02	chrX	MIMAT0000457	49768132	49768143	-
miR-122	2.00±1.58	0.13	chr18	MIMAT0000421	56118324	56118341	-
miR-877*^2^	1.83±0.80	0.10	chr6	MIMAT0004950	30552182	30552194	-
miR-146a	1.71±0.78	0.03	chr5	MIMAT0000449	1.6E+08	1.6E+08	-
miR-548q	1.68±0.57	0.04	chr10	MIMAT0011163	12767281	12767267	+
miR-20a	1.65±0.57	0.05	chr13	MIMAT0000075	92003331	92003348	-
miR-125a-3p	1.65±0.14	0.04	chr19	MIMAT0004602	52196565	52196580	-
miR-32*	1.63±0.76	0.15	chr9	MIMAT0004505	1.12E+08	1.12E+08	+

1, chr: chromosome;

2, *: minus.

### miR-572 expression changes were associated with cognitive function restoration in the POCD rat model

**Fig 2:**
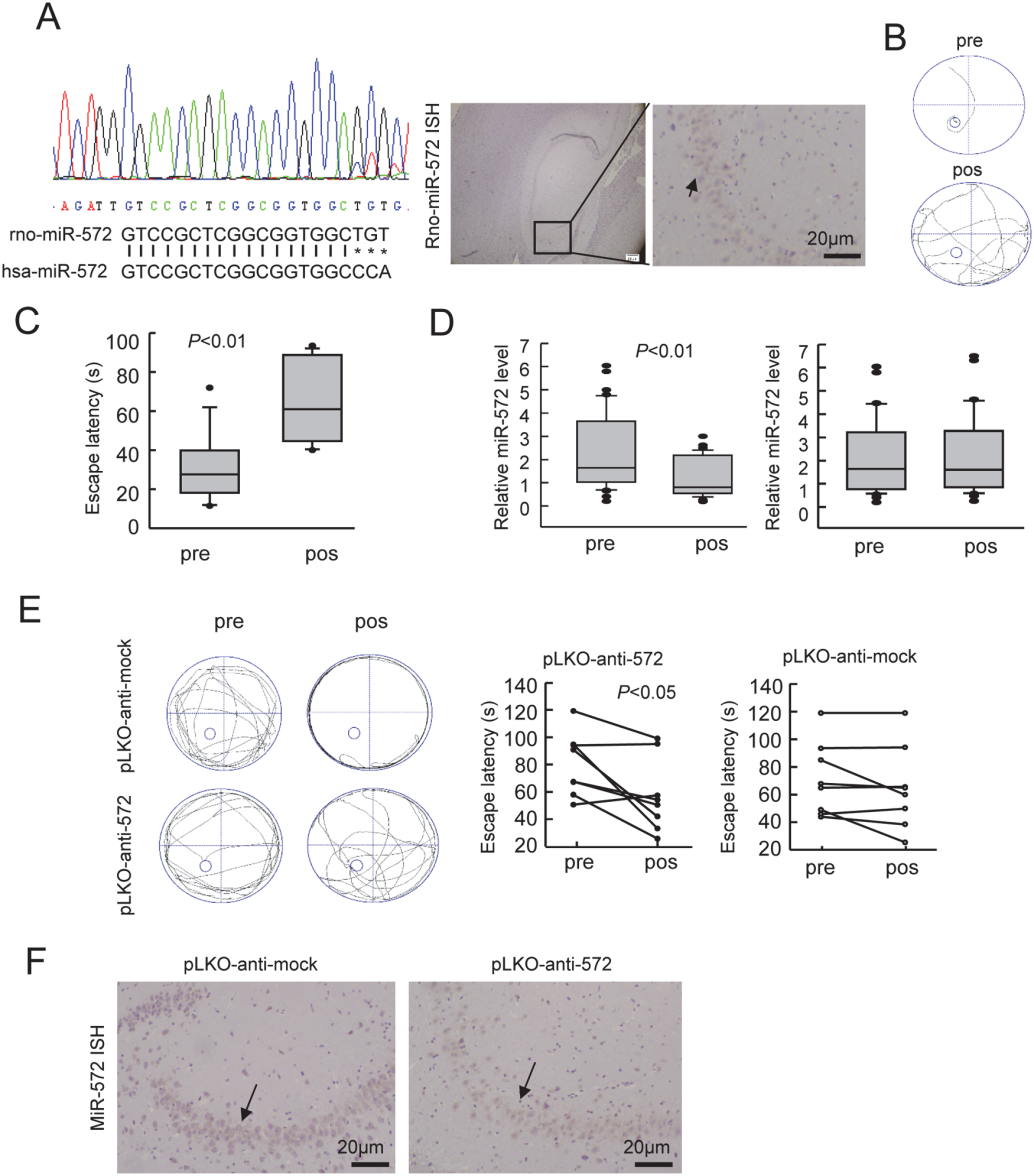
Effects of miR-572 on the cognitive function of the POCD rat model. A. Detection of miR-572 in rat peripheral blood (left) and in situ hybridization detection of miR-572 expression in the rat hippocampus area. B. Schematics of the water maze routes. C. After surgery, the POCD rats had a longer latency in finding the platform in the water maze test compared to the time needed before surgery. D. Real-time quantitative PCR detection of miR-572 in the peripheral blood of POCD rats and non-POCD rats before and after surgery. In POCD rats, the postoperative miR-572 expression in the peripheral blood was lower than the preoperative level (left), while the non-POCD rats did not show significant changes in the miR-572 expression before and after surgery (right). E. Schematic diagram of the water maze route for the POCD rats after treatment with the miR-572 inhibitor (left). After treatment, the latency of the rats in the water maze test was significantly reduced (middle), whereas the control group showed no significant improvement (right). F. In situ hybridization detection found that after injection of inhibitors, the expression level of miR-572 in the rat hippocampus was decreased. pre, preoperation; pos, postoperation.

POCD patients showed significantly impaired cognitive function after surgery and had reduced miR-572 expression level in the postoperative peripheral blood, suggesting that miR-572 may be involved in maintaining the cognitive function of patients. To further clarify whether miR-572 was involved in the improvement of postoperative cognitive function, we established a rat model of POCD for in vivo functional tests. Because miR-572 expression in the rat has not been reported so far, we first examined whether there is a rat miRNA molecule homologous to hsa-miR-572. We used the primer sequences for hsa-miR-572 (Table 2) to perform PCR and sequencing analyses to demonstrate the existence of an miRNA sequence in the peripheral blood of rats that has an 85% similarity to hsa-miR-572 (Fig. 2A *left*), and named it rno-miR-572. Moreover, we used in situ hybridization to detect rno-miR-572 expression in the rat hippocampal tissues (Fig. 2A *right*). Using the method reported by Barrientos et al. we performed a splenectomy in aging rats. The water maze test illustrated that, compared with before surgery, the latency of boarding the platform of animals receiving a splenectomy was significantly prolonged (Fig. 2B, 2C), suggesting successful establishment of a rat model of POCD. Real-time PCR found that, compared with the pre-operative level, the miR-572 expression level in the post-operative peripheral blood was significantly reduced (Fig. 2D *left*). In addition, rats without cognitive dysfunction showed no significant changes of miR-572 expression (Fig. 2D *right*). The results were consistent with those of clinical specimens, suggesting that miR-572 might be involved in regulating cognitive function changes in the POCD rat model. Because miRNAs mainly play a negative regulatory role, we hypothesized that in the early stage of POCD, the body might down-regulate miR-572 to up-regulate its downstream target genes, thus contributing to the restoration of cognitive function. Therefore, we selected rats with reduced cognitive function and used a lateral ventricular micropump to inject the miR-572 inhibitor to further validate the above hypothesis. After 3 days, the water maze test found that, compared with the control (pLKO-anti-mock), rats injected with pLKO-anti-572 showed significantly shorter latency to board the platform, suggesting significantly improved cognitive function (Fig. 2E). Using in situ hybridization analysis, we found that the miR-572 expression in the brain tissue was decreased after the injection of pLKO-anti-572 (Fig. 2F). The above results indicate that miR-572 can participate in maintaining the cognitive function of rats and that inhibition of miR-572 expression can improve the cognitive function of POCD rats.

### Mechanism of miR-572-mediated restoration of postoperative cognitive function

**Fig 3:**
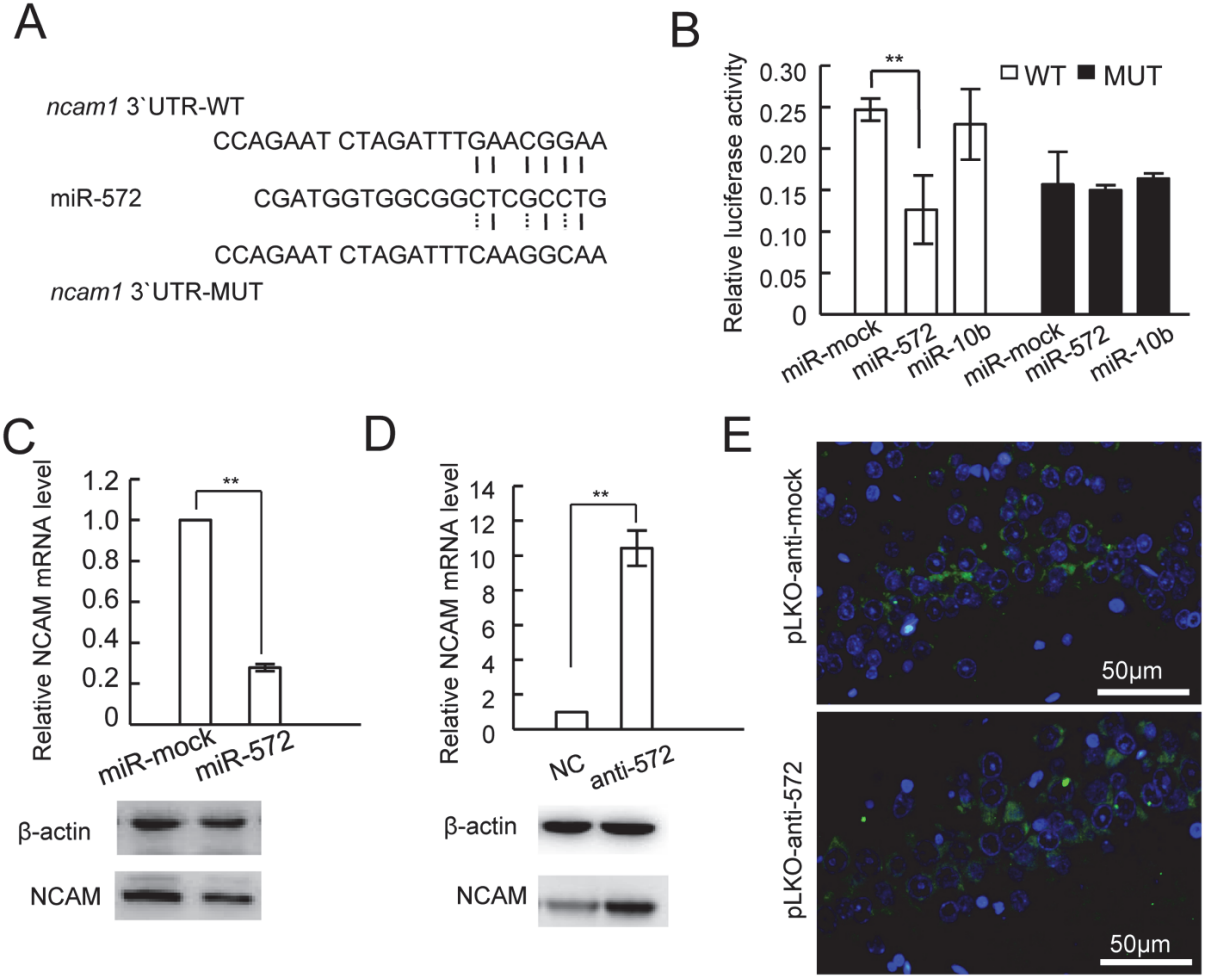
Targeted regulation of the expression of NCAM by miR-572. A. Schematics of miR-572 binding to the 3'UTR region of the NCAM1 mRNA (wildtype and mutant) in the dual-luciferase experiment. B. The dual-luciferase assay showed that miR-572 significantly reduced the luciferase activity of plasmids containing the wildtype 3'UTR region of mouse NCAM1 mRNA. C. Overexpression of miR-572 in mouse HT22 cells could significantly reduce the NCAM1 expression at the mRNA and protein levels. D. Inhibition of miR-572 in mouse HT22 cells could significantly promote NCAM1 expression at the mRNA and protein levels. E. Immunohistochemical detection showed that after inhibiting miR-572 expression in the POCD rat brain, the NCAM1 expression was elevated. WT, wildtype; MUT, mutant; NC, negative control.

Next, we went further to investigate the potential molecular mechanism by which miR-572 was involved in the restoration of cognitive function. We used bioinformatic analysis to identify the potential genes downstream of miR-572 (TargetScan). The results suggested that hsa-miR-572 has 9 potential downstream target genes (Table 4). Among them, neural cell adhesion molecule 1 (NCAM1) plays an important role in improving neuronal cell variability and increasing axonal proliferation, neuronal plasticity, and cognitive function [28]. Therefore, we hypothesized that miR-572 might participate in the restoration of cognitive function by regulating the expression of NCAM1. To verify whether miR-572 regulates the expression of NCAM1 by binding to the 3' untranslated region (3'UTR) of NCAM1, we first constructed a luciferase plasmid containing the 3'UTR of the NCAM1 mRNA, which contained the potential miR-572 binding sites (pMir-NCAM1-UTR-WT) and a plasmid containing mutated binding sites (pMir-NCAM1-UTR-MUT) (Fig. 3A). miR-572 and the luciferase plasmids were co-transfected into the mouse hippocampal neuronal cell line HT22, and the dual luciferase analysis showed that miR-572 significantly reduced the luciferase activity of pMir-NCAM1-UTR-WT; however, the luciferase activity of pMir-NCAM1-UTR-MUT was not significantly changed (Fig. 3B). This result suggests that miR-572 is capable of binding to the 3'UTR region of NCAM1. To test whether miR-572 can regulate the expression of NCAM1, we used real-time quantitative PCR and western blotting analysis to detect the NCAM1 expression in HT22 cells transiently transfected with the miR-572 mimic and found that miR-572 could inhibit the NCAM1 expression at both the mRNA and protein levels (Fig. 3C). When miR-572 was down-regulated by the transfection of the miR-572 inhibitor, the NCAM1 expression was increased (Fig. 3D). This finding suggests that miR-572 can regulate the expression of NCAM1 in hippocampal neurons. In addition, immunohistochemical analysis revealed that injections of pLKO-anti-572 into the brain tissues of rats with cognitive dysfunction could promote NCAM1 expression (Fig. 3E). These results suggest that miR-572 can regulate the NCAM1 expression in neuronal cells.

**Table 4:** Candidate Genes for miR-572 Downstream Targets

Target gene	Gene name	Total context score
FLJ45910	FLJ45910 protein	-0.42
C14orf101	chromosome 14 open reading frame 101	-0.31
NCAM1	neural cell adhesion molecule 1	-0.23
SAP30BP	SAP30 binding protein	-0.2
ADRBK1	adrenergic, beta, receptor kinase 1	-0.16
VSTM2A	V-set and transmembrane domain containing 2A	-0.13
RAB35	RAB35, member RAS oncogene family	-0.08
NFIX	nuclear factor I/X (CCAAT-binding transcription factor)	-0.07
MECP2	methyl CpG binding protein 2 (Rett syndrome)	0

### Clinical correlation analysis of miR-572

**Fig 4:**
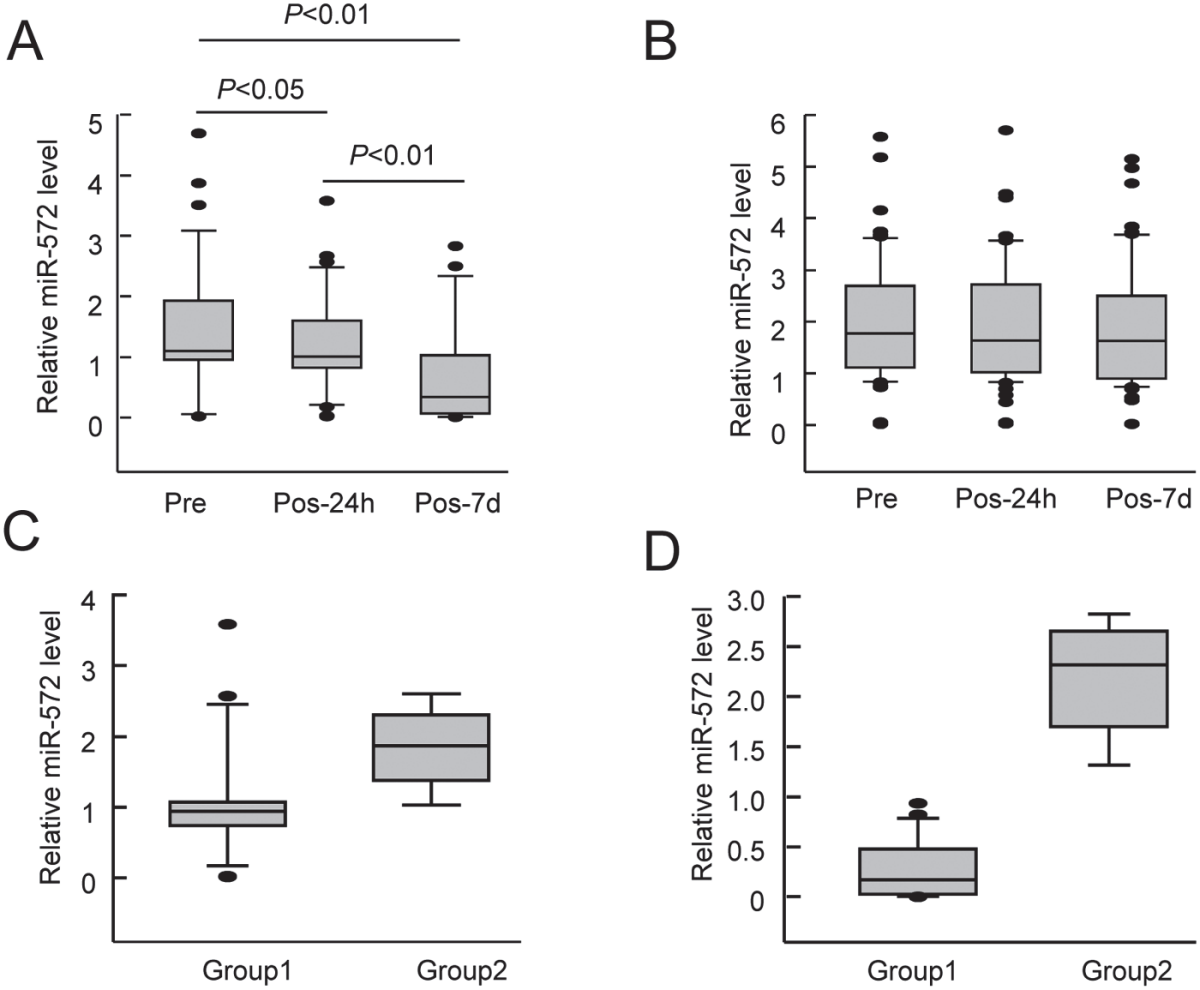
miR-572 expression in the peripheral blood of clinical patients. A. Real-time quantitative PCR detection of the miR-572 expression levels in the peripheral blood of POCD patients (n = 38) before surgery (Pre), 24 h after surgery (Pos-24h), and 7 days after surgery (Pos-7d). B. Real-time quantitative PCR detection of the miR-572 expression levels in the peripheral blood of non-POCD patients (n = 62) before surgery (Pre), 24 h after surgery (Pos-24h), and 7 days after surgery (Pos-7d). C. Real-time quantitative PCR detection of the miR-572 expression levels in the peripheral blood of POCD patients whose cognitive function was recovered 3 months after surgery (n = 29, Group1) and whose cognitive function was not recovered (n = 9, Group2) 24 h after surgery. D. Real-time quantitative PCR detection of the miR-572 expression levels in the peripheral blood of POCD patients whose cognitive function was recovered 3 months after surgery (n = 29, Group1) and whose cognitive function was not recovered (n = 9, Group2) 7 days after surgery.

To test whether miR-572 can be used as a marker for the early diagnosis and prognosis of POCD, we collected the peripheral blood from elderly POCD patients (n = 38) before and 24 h and 7 days after surgery. Real-time quantitative PCR was used to measure the miR-572 expression in the peripheral blood samples. The results showed that the blood miR-572 levels in POCD patients (n = 38) at 24 h (1.21 ± 0.79) and 7 days (0.724 ± 0.883) after the surgery were significantly lower than the preoperative level (1.42 ± 1.06) (Fig. 4A). The peripheral blood miR-572 levels of the non-POCD patients (n = 62) at 24 h and 7 days after the surgery (1.92 ± 1.16 and 1.95 ± 1.30, respectively) had no significant change compared with the preoperative level (2.03 ± 1.15) (Fig. 4B). It suggested the correlation between miR-572 expression and POCD.

For further analysis, Mini Mental State Examination (MMSE) was used to assess the cognitive function of patients with POCD. We found that the performances of most patients (n = 29, Group1) restored to the preoperative level (MMSE score 26.14±2.56 vs 26.48±2.29, *P*>0.05) in the neurocognitive function tests at 3 months after the surgery. Nine patients (Group 2) failed to restore the preoperative level and had varying degrees of residual cognitive impairments (MMSE score 21.11±1.94 vs 26.56±2.13, *P*< 0.01). Quantitative PCR showed that the blood miR-572 expression levels in these 9 patients were significantly higher at 24 h and 7 days after the surgery than those of the other POCD patients (Fig. 4C, 4D). These results suggest that the miR-572 expression levels of POCD patients may affect the restoration of postoperative cognitive function.

## Discussion

In this study we found that POCD patients had an abnormal miRNA expression in the peripheral blood after surgery. Among these altered miRNAs, the expression of miR-572, which is involved in the repair of cognitive function, was down-regulated. After surgery, the decline in cognitive function was accompanied by decreased expression of miR-572 in the peripheral blood and hippocampal region. Interfering with miR-572 expression in rats can facilitate the restoration of cognitive function in the rats. Furthermore, in hippocampal neurons, miR-572 can regulate the expression of NCAM1. The abnormal changes of miR-572 in peripheral blood may be used as an auxiliary diagnostic marker for the early diagnosis of POCD and prediction of POCD prognosis.

Clinically, cognitive function can recover to different degrees in some POCD patients in a given period. Our research explored the molecular mechanisms underlying the cognitive function recovery. It has been reported that NCAM1 can improve the synaptic plasticity of the hippocampal region, improving the variability of neurons, repairing degenerated neurons, and improving cognition, learning, and memory capacity [29]. Our study found that in rat POCD model inhibiting miR-572 expression could up-regulate NCAM1 expression and improve cognitive function in rats, suggesting that increased NCAM1 expression may vital for the restoration of cognitive function in POCD patients. It is possible that when the nervous system is damaged to the extent which causing cognitive impairment, miR-572 expression will be down-regulated to promote NCAM1 expression, initiating the repair mechanisms to promote cognitive function restoration.

Some POCD patients present with a permanent cognitive dysfunction-dementia. Studies have suggested that permanent POCD is related to neuronal degeneration and death in the central nervous system. In our study, the 9 POCD patients whose neurocognitive function did not return to the preoperative level at 3 months after surgery had a higher postoperative blood miR-572 were higher than that of patients whose neurocognitive function returned to the normal level, which might be due to the failed initiation or inhibition of the repair process, that is, increased miR-572 expression leads to reduced downstream NCAM1 expression and loss of neuronal protection, resulting in degeneration and necrosis of the neurons. These results confirm the important role of NCAM1 in neural repair and also suggest that a postoperative adjuvant therapy targeting NCAM1 in POCD patients may help to prevent cognitive impairment from developing into dementia. Our findings cast new light on the treatment of late stage POCD. However, the specific mechanism remains to be further investigated.

miR-572 expression was observed to have an evident correlation with the incidence of POCD, and miR-572 could reflect the pathogenic conditions of POCD patients in early stages and could serve as a biological marker for the early diagnosis of POCD. In addition, we found that in patients whose neurocognitive function did not return to the preoperative level by 3 months after surgery, the postoperative blood miR-572 expression was higher than that of patients whose neurocognitive function test returned to the normal. In clinical practice, to distinguish the postoperative cognitive dysfunction caused by the residual action of postoperative anesthetics, postoperative pain, changes in the postoperative sleep rhythm, and mental disorders caused by surgical trauma such as anxiety and depression, examinations of the patient’s clinical symptoms and related neurocognitive function tests are performed to POCD diagnosis at 7 days after surgery, with other early corresponding quantitative detection indicators not available at present [30]. However, to reveal the in-depth molecular mechanism of cognitive function restoration in patients with POCD, more research is required as follows: first, the regulatory mechanism of miR-572 expression after the onset of POCD needs to be revealed, including the reason why miR-572 expression was higher in patients whose neurological function did not recover to normal by 3 months after surgery than that in those returned to the normal; second, the impact of miR-572 on the biological function of hippocampal neurons needs to be evaluated both in vitro and in vivo; and third, POCD diagnosis, auxiliary treatment drugs, and other related research for clinical application also need further study. And related studies are currently being performed in our laboratory.

## Supporting Information

S1 ARRIVE Checklist (PDF)

S1 Table Clinicopathologic Features of POCD Patients. (DOC)
