# Supplementary material for: MicroRNA-572 Improves Early Post-Operative Cognitive Dysfunction by Down-Regulating Neural Cell Adhesion Molecule 1
Source: PLoS One. 2015 Feb 13;10(2):e0118511. doi: 10.1371/journal.pone.0118511 (PMC4334516; doi:10.1371/journal.pone.0118511)
Supplement: S1 Table — (DOC) [file pone.0118511.s002.doc]

**Table S1**. **Clinicopathologic Features of POCD Patients**

| **Variable** | **Patients with restoration of cognitive function**  **(n=29)** | **Patients without restoration of cognitive function**  **(n=9)** |  |
| --- | --- | --- | --- |
| Age  Mean(S.D) | 71.14(5.16) | 73.11(7.84) |  |
| Gender | | | |
| Male | 6 (20.7%) | 4 (44.4%) |  |
| Female | 23 (79.3%) | 5 (55.6%) |  |
| ASA |  |  |  |
| II | 29 (100%) | 9 (100%) |  |
| MMSE  Mean(S.D) |  |  |  |
| pre | 26.48 (2.29) | 26.56 (2.13) |  |
| post-3M | 26.14 (2.56) | 21.11 (1.94) |  |

ASA, American Society of Anesthesiologists physical status; MMSE, Mini Mental State Examination; pre, preoperation; post-3M, postoperation 3 months
